# Case Report: Refusal of an Veno-Arterial Extracorporeal Membrane Oxygenation Due to Malignant Disease? — An Extremely Rare Form of Cardiac Involvement in Acute Myeloid Leukemia

**DOI:** 10.3389/fmed.2021.584507

**Published:** 2021-02-23

**Authors:** Viviane Zotzmann, Tobias Wengenmayer, Corinna N. Lang, Dawid L. Staudacher, Katharina Mueller-Peltzer, Fabian Bamberg, Reinhard Marks, Christoph Bode, Ralph Wäsch

**Affiliations:** ^1^Department of Medicine III (Interdisciplinary Medical Intensive Care), Medical Center, Faculty of Medicine, University of Freiburg, Freiburg im Breisgau, Germany; ^2^Department of Cardiology and Angiology I, University Heart Center Freiburg, Bad Krozingen, Germany; ^3^Department of Diagnostic and Interventional Radiology, Faculty of Medicine, University of Freiburg, Freiburg im Breisgau, Germany; ^4^Department of Medicine I (Hematology, Oncology, and Stem-Cell Transplantation), Faculty of Medicine, Medical Center, Freiburg im Breisgau, Germany

**Keywords:** extramedullary acute myeloid leukemia, cardiac myeloid sarcoma, veno-arterial extracorporeal membrane oxygenation, right heart failure, ECLS, AML, VA-ECMO

## Abstract

We report here on a 61-year-old patient with acute right heart failure of unclear etiology. Echocardiography revealed a myocardial mass infiltrating the heart, though, we assumed a cardiac lymphoma. A VA-ECMO was implanted as bridging for diagnosis and therapy. Our patient received chemotherapy, under which the tumor (of unknown etiology at this point) reached a partial remission. Nine months after first admission the patient developed acute myeloid leukemia with *DNMT3a* and *TET2* mutations. Retrospective analysis of the cardiac biopsy revealed the identical mutations and matched with the diagnosis of an extremely rare primary extramedullary manifestation of an AML (myelosarcoma). The patient received induction-chemotherapy and was planned for consolidating allogeneic stem cell transplantation. From this case, we conclude that an extracorporeal therapy should be discussed in selected patients even in case of an initially fatal appearing prognosis. In selected cases, extracorporeal support can generate enough time for diagnosis and therapy. However, transparent planning, including discussion of best supportive care strategies involving the patient's family are indispensable requirements for starting ECMO in such patients.

## Introduction

Veno-arterial extracorporeal membrane oxygenation (VA-ECMO) describes a method for cardiopulmonary bypass using of a pump-driven circulation, in which venous deoxygenated blood is extracorporeally enriched with oxygen. It is indicated for potentially reversible, life-threatening forms of cardiac failure as “bridge to recovery,” “bridge to next decision” or “bridge to transplant or assist device” (1). There is hardly any data on the implantation of VA-ECMO in adult cancer patients (2–4).

It is well-known that prolonged use of ECMO support increases the risk of morbidity, mostly due to thrombotic, and hemorrhagic complications (5). Nosocomial infections may even be triggered by impairment of cellular immunity, cytopenia and chemotherapy, and may dis- suade clinicians from using ECMO support in these patients (6, 7).

More precisely, malignancies are regarded as a relative contraindication for VA-ECMO support and the extracorporeal therapy community shows utmost restraint in applying extracorporeal therapy for cancer patients. Some authors argue that implementation of ECMO support should only be taken into consideration in cases for whom a 100% mortality would be expected with lower-level interventions (8).

## Case Report

### Medical History

**Figure 1:**
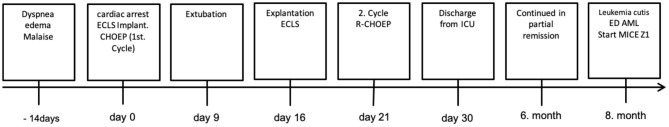
Timeline: patient's course over time.

A 61-year-old man without any pre-existing conditions was admitted to our center with malaise, night sweat, a weight loss of 11 kg over the last 6 months, and increasing dyspnea for 2 weeks (Figure 1).

### Clinical Course

**Figure 2:**
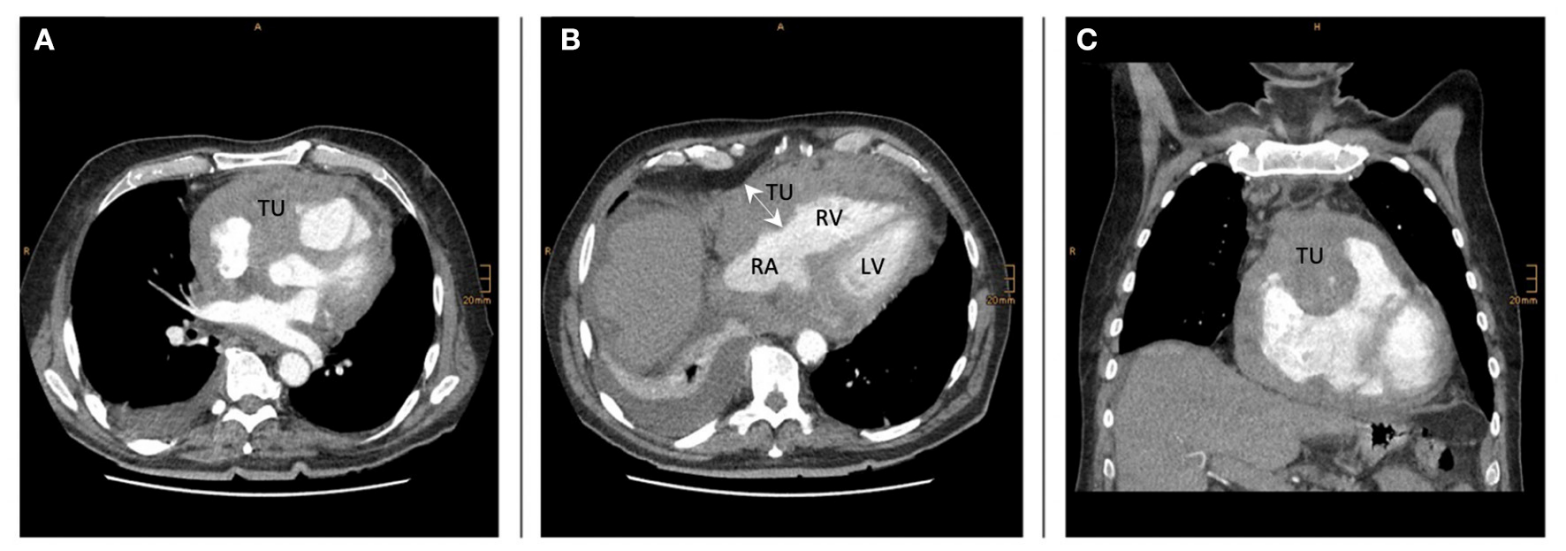
TU tumor mass-infiltration in the right ventricular wall and around the aortic root (**A,B** shows the heart and tumor mass in transversal view; **C** sagittal view on the heart and the tumor-formation) RA, right atrium; RV, right ventricle; LV, left ventricle.

He presented with shortness of breath (NYHA III) and pronounced edema of both legs. The ECG showed atrial fibrillation, the X-ray of the chest a pulmovenous congestion and a large pleural effusion. Abdominal and thoracic CT-scan (Figure 2) showed multiple mediastinal and mesenterial lymph nodes, a bilateral perirenal mass and a large pericardial lymph node conglomerate with infiltration of the right atrium, ventricle, and parts of the aorta ascendens. Blood smear and flow cytometry of the peripheral blood (PB) and bone marrow (BM) biopsy appeared normal without indication for a hematological neoplasia.

**Figure 3:**
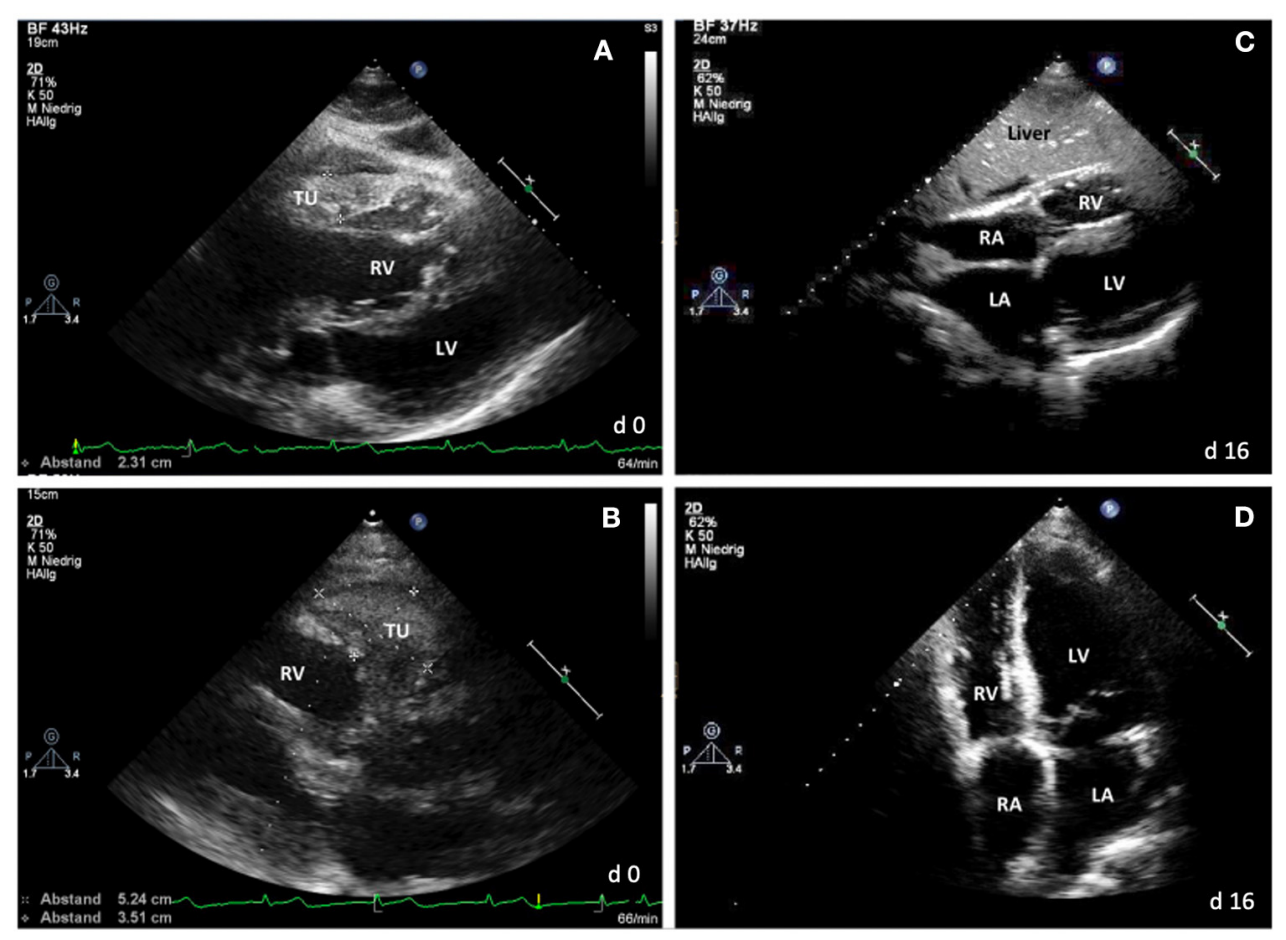
Echocardiography. **(A,B)** At day 0: showing a pericardial tumor and hypertrophy of the right ventricle wall. **(C,D)** At day 16: showing no hypertrophy or tumor mass any more. TU, tumor mass; RA, right atrium; RV, right ventricle; LA, left atrium; LV, left ventricle.

Echocardiography (Figure 3) showed a severe impairment of the right ventricle due to a pronounced hypertrophy of the free right ventricular wall and a tumor (50 × 60 mm) right next to the right ventricular wall. These findings corresponded to the patient's severe right heart failure symptoms. Before being able to perform a mini-laparotomy for analyzing an abdominal lymph node, the patient experienced a cardiac arrest, had to be resuscitated, and was transferred to our ICU.

Despite high doses of catecholamine's and volume resuscitation hemodynamics could not be stabilized (increasing lactat level 17 mmol/L, SAPS II 64, SAFE score −6). Assuming a lymphoma-infiltration of the right ventricle as a potentially curative disease a VA-ECMO was implanted as a “bridge to diagnostic and therapy.”

In suspicion of a lymphoma-infiltration as cause for the life-threatening right heart failure, we decided to immediately start chemotherapy the same day—CHOEP without Doxorubicin due to cardio-toxicity.

After clinical stabilization, a myocardial biopsy and an abdominal lymph node extirpation were performed while still on VA-ECMO support. There were no signs of myocarditis and histology and immunohistochemistry showed no lymphocytic infiltration of the myocardium and only reactive changes of the lymph node, most likely due to the chemotherapy. Consistent with this observation lymph nodes, pericardial tumor and hypertrophy of the right ventricle wall were rapidly regressive and the right heart function improved dramatically during the chemotherapy course.

Nine days later the patient could be weaned from the respirator, after 10 days he recovered from renal failure and after 16 days VA-ECMO could be explanted.

The course was complicated by an Aspergillus pneumoniae during neutropenia and had to be treated for 23 days. Due to the good response with regress of the cardiac infiltration and decline of the abdominal lymph nodes, the patient received the second cycle including rituximab (R-CHOEP), at day 21.

A CT-Scan (4 weeks later) showed a partial remission of the tumors. The tumor-mass around the aorta ascendens, near the right ventricle and at the upper kidney pole showed decreased diameters. He could be discharged from hospital 7 weeks after admission.

In the following months, the patient received four more cycles of chemotherapy, under which the tumor was kept stable in a partial remission.

Eight month after the first admission he was again admitted to our hospital after a dermal biopsy showed a diffuse dermal infiltration of immature myeloid cells (70%). BM biopsy now confirmed an acute myeloid leukemia (AML) with a blast count of 70–80% and suppression of the normal hematopoiesis. Molecular genetic testing revealed mutations in NPM1, DNMT3a and TET2. These results prompted us to search for these mutations in the initial cardiac biopsy. Indeed, the DNMT3A-mutation and the TET2-mutation were detected. Retrospectively, the initial infiltration of the right ventricle wall matched with a myeloid sarcoma. Nine months after first admission the patient received induction chemotherapy with a planned allogeneic stem cell transplantation for consolidation.

## Discussion

### Cardiac Involvement of Lymphomas and Leukemia

The incidence of primary cardiac tumors is rare, ranging from 0.001 to 0.03% in autopsies (9, 10). The most prevalent primary malignant tumors are cardiac sarcomas, of which angiosarcoma is the most common in adults (37%) (11). Among primary cardiac malignancies lymphomas are extremely rare (1–2%) and are found especially in immunosuppressed patients (12). They are mostly Non-Hodgkin-B cell type and preferentially involve the right atrium (6).

A secondary involvement of the heart has been seen in 8.7–27.2% of the lymphoma patients (12). Prognosis of patients with cardiac tumors is in general poor. Mean overall survival ranges from 1 to 12 months (9, 13). However, in patients with cardiac lymphoma and adequate treatment long-term survival can be reached.

AML may present in a variety of extramedullary tissues with or without bone marrow disease. Extramedullary involvement is a relatively rare, but clinically significant phenomenon that often poses therapeutic dilemmas. Myeloid sarcoma (MS) and leukemia cutis (LC) represent two extramedullary manifestations. MS is reported in 2.5–9.1% of patients with AML (14, 15) and occurs concomitantly, following, or rarely, preceding the onset of bone marrow involvement (16).

Isolated MS, defined by the absence of a history of leukemia, MDS, or myeloproliferative neoplasm and a negative bone marrow biopsy, has been described in very few case reports (17).

In a series reporting autopsies of AML patients, involvement of the myocardium with MS was found to occur in <1% of the patients (18). Many of these patients are often misdiagnosed with lymphoma (19, 20). Generally, patients with MS are considered as having high-risk AML with a poor prognosis.

### Ethical, Medical, and Technical Challenge

Our patient presented with a worsening, severe cardiogenic shock with right heart failure due to a tumorous infiltration of the right myocardium. Without extracorporeal support, this patient would have probably died in the next 24 h. However, numerous medical and ethical challenges may arise when installing VA-ECMO in patients with known malignancy.

For instance, the Extracorporeal Life Support Organization (ELSO) lists contraindications for applying extracorporeal support in patients such as chronic organ dysfunction like emphysema, cirrhosis, or renal failure. Moreover, an unrecoverable heart condition is an absolute contraindication for VA-ECMO support *per se* when the patient is no candidate for a permanent mechanical assist device. In our case, etiology of right heart failure was unclear, however, we assumed a potentially curable cardiac lymphoma.

Bleeding complications, arterial embolism and limb ischemia are typical complications of a VA-ECMO therapy. In particular, the thrombocytemia to be expected under chemotherapy and the increased risk of bleeding with an already known intracerebral bleeding rate of 21% under ECMO therapy in non-cancer patients (21) represent a high risk for the patient (relative contraindication).

Additionally, ECMO support is associated with nosocomial infections (6). The impairment of cellular immunity, cytopenia, and chemotherapy may further increase the risks of infection.

ECMO support is associated with high rates of mortality *per se*. It is important to understand that the extracorporeal support does not support or specifically treat the heart itself. It just delivers the amount of extracorporeal cardiac output to maintain organ function. However, patients with chronic organ dysfunctions are less likely to survive profound shock situations, for that some very invasive procedures are withheld from them after individual considerations. ECMO support is a highly invasive, cost and resource intense therapy and comes with the cost of serious side effects. From this point of view, it is reasonable that chronic organ failure is a relative contraindication for ECMO support.

Our patient presented without chronic organ failure, but with an undiagnosed, and untreated disease.

Due to the known risks and the relative contraindications, the published experience with VA-ECMO in children and adult patients with hematologic malignancies is limited to some single cases and a registry analysis (2–4, 22). One of these cases is about a child who suffered cardiac arrest when first diagnosed with T-lymphoblastic lymphoma that infiltrates the heart.

After implantation of a VA-ECMO, chemotherapy was carried out under ECMO support and the patient was finally able to be discharged home on the 28th day. This case was able to demonstrate the usefulness and feasibility of chemotherapy while on ECMO-support (22).

After in-depth discussion with the patient's family, we decided to implant the VA-ECMO with the aim of “bridge to diagnosis and therapy” assuming a potentially curable disease.

## Conclusion

ECMO has become a lifesaving therapy in patients with severe cardiac impairment. From this case, we conclude that an extracorporeal therapy should at least be discussed in selected patients even in case of an initially fatal appearing prognosis. Once extracorporeal support is installed a thoroughly but fast diagnostic workup has to be achieved to enable a therapy start as soon as possible.

In selected cases extracorporeal support can generate valuable days of diagnosis and therapy. However, transparent planning, including discussion of best supportive care strategies involving the patient's family are indispensable requirements for starting such a therapy.

## Data Availability Statement

The raw data supporting the conclusions of this article will be made available by the authors, without undue reservation.

## Ethics Statement

Ethical review and approval was not required for the study on human participants in accordance with the local legislation and institutional requirements. The patients/participants provided their written informed consent to participate in this study.

## Consent for Publication

The patient himself gave his oral consent and his wife the written consent for publication to the treatment team.

## Author Contributions

VZ was the major contributor in writing the manuscript. CL and CB revised the manuscript. KM-P created the figures. FB revised the manuscript. TW and DS designed figures and revised the manuscript. RM and RW revised the manuscript. All authors read and approved the final manuscript.

## Conflict of Interest

The authors declare that the research was conducted in the absence of any commercial or financial relationships that could be construed as a potential conflict of interest.

## Abbreviations

RV: right ventricle
LV: left ventricle
RA: right atrium
LA: left atrium
TU: tumor mass
LI: liver
PE: pericardial effusion
PB: peripheral blood
BM: bone marrow
VA-ECMO: veno-arterial extracorporeal membrane oxygenation
ELSO: extracorporeal life support organization
ECLS: extracorporeal life support
R-CHOEP, Chemotherapy including rituximab, cyclophosphamid, doxorubicin, vincristin, etoposid: prednisolone.
